# Pan‐ERBB Inhibitors Synergize With KRAS Inhibitors in Rectal Cancer

**DOI:** 10.1002/ueg2.70086

**Published:** 2025-09-16

**Authors:** Jonas Buchloh, Melanie Spitzner, Hauke Zimmermann, Xin Fang, Constanza Tapia Contreras, Carolin Schneider, Tiago de Oliveria, Stefan Küffer, Michael Linnebacher, Felix Rühlmann, Lena Conradi, Matthias Wirth, Michael Ghadimi, Marian Grade, Jochen Gaedcke, Günter Schneider

**Affiliations:** ^1^ Department of General, Visceral and Pediatric Surgery University Medical Center Göttingen Göttingen Germany; ^2^ Institute of Pathology University Medical Center Göttingen University of Göttingen Göttingen Germany; ^3^ Molecular Oncology and Immunotherapy Clinic of General Surgery University Medical Center Rostock Rostock Germany; ^4^ Clinic für General‐ and Visceral Surgery Städtisches Klinikum Karlsruhe Karlsruhe Germany; ^5^ CCC‐N (Comprehensive Cancer Center Lower Saxony) Göttingen Germany; ^6^ Department of Hematology, Oncology and Cancer Immunology Campus Benjamin Franklin Charité—Universitätsmedizin Berlin Corporate Member of Freie Universität Berlin and Humboldt‐Universität zu Berlin Berlin Germany; ^7^ Institute for Translational Cancer Research and Experimental Cancer Therapy Technical University Munich Munich Germany

**Keywords:** disease‐free survival, drug resistance, EGFR, ERBB, KRAS, RAS, rectal cancer, therapy response, transcriptomic

## Abstract

**Background:**

Emerging RAS inhibitors show promise in treating KRAS‐mutated malignancies, but resistance mechanisms limit their clinical efficacy. Given recent clinical findings associating KRAS mutations with reduced response to neoadjuvant therapy in rectal cancer (RC), we aimed to investigate their impact on treatment outcomes and explore potential therapeutic strategies.

**Methods:**

We conducted a retrospective analysis of 390 rectal cancer patients to evaluate the association of KRAS mutations with disease‐free survival (DFS) and response to therapy. We assessed the efficacy of KRAS inhibitors in rectal cancer cell lines, patient‐derived organoids (PDOs), and patient‐derived cell lines (PDCLs), and explored adaptive resistance mechanisms through transcriptomic profiling and unbiased drug screening experiments.

**Results:**

Mutant KRAS was associated with a reduced DFS and RCs harboring G12C and G12V mutations had less complete pathological responses to neo‐adjuvant therapies. KRAS‐mutated RC cells demonstrated adaptive resistance to KRAS inhibitors, characterized by transcriptomic restoration of oncogenic pathways, including MYC and E2F, and upregulation of ERBB2/3 expression. Consistently, drug screening identified EGFR family inhibitors as potent combinatorial partners, effectively overcoming KRAS inhibitor tolerance by inducing apoptosis. In patient‐derived models, the pan‐RAS inhibitor RMC‐6236 combined with EGFR inhibitors demonstrated significant synergistic effects and prevented long‐term tumor cell outgrowth.

**Conclusion:**

Our findings point to the negative impact of KRAS mutations, particularly G12C and G12V, on RC treatment outcomes. Adaptive resistance by upregulation of ERBB genes limits the efficacy of KRAS inhibitors. Combining these with pan‐ERBB inhibitors enhances anti‐tumor effects in patient‐derived cellular RC models, showing its potential as an alternative to the combination with anti‐EGFR antibodies.

## Key Summary

- Summarize the established knowledge on this subject
  ◦ Locally advanced rectal cancer is treated with multimodal therapy, including total neoadjuvant therapy (TNT)
  ◦ Response rates to total neoadjuvant therapy (TNT) vary, underscoring the need for predictive biomarkers and alternative treatment strategies for non‐responders.
- What are the significant and/or new findings of this study?
  ◦ In retrospective investigations, oncogenic KRAS mutations, particularly the G12C and G12V variants, were associated with both reduced disease‐free survival and diminished complete pathological response rates.
  ◦ KRAS inhibitors (KRASi) induce an early adaptive response limiting their efficacy.
  ◦ In patient‐derived rectal cancer models, inhibition of the EGFR family, including ERBB2/3, improves KRASi response, and delays resistance emergence.

## Graphical abstract

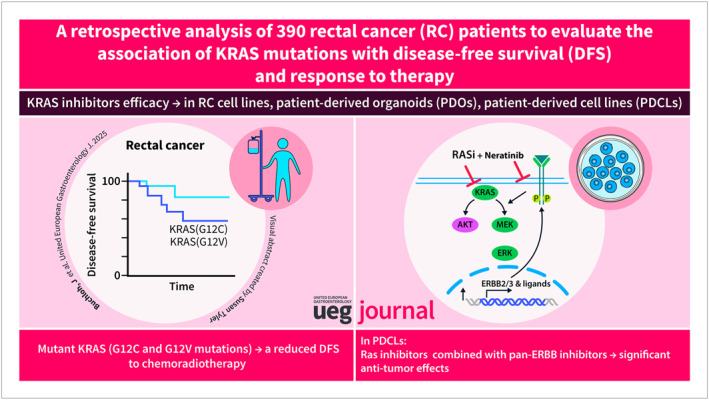

## 1 Introduction

Rectal cancer (RC) accounts for approximately one‐third of all CRC cases. Notably, particularly in younger patients under the age of 50, the incidence of RC is increasing [1]. Multimodal therapy for patients with locally advanced RC involves neoadjuvant chemoradiotherapy (CRT) followed by surgery [2]. With the aim of early treating micro‐metastatic disease, reducing the risk of distant failure, and improving local control, total neoadjuvant therapy (TNT) seeks to combine all systemic therapy and CRT upfront from surgery, with the option of a watch‐and‐wait strategy for patients with a clinical complete response [3]. Variable response rates to TNT highlight the need for response biomarkers and alternative therapies to improve treatment outcomes to allow personalized treatments [4].

Rat sarcoma viral oncogene (RAS) gene mutations are common in cancer, affecting approximately 19% of all cancer cases. The Kirsten rat sarcoma virus (KRAS) gene is the most frequently mutated RAS gene, accounting for approximately 75% of all RAS mutations [5]. KRAS alterations occur in 45% of colorectal cancers (CRC) with the G12D mutation dominating [6]. After decades of preclinical research, RAS inhibitors have entered the clinic, with KRAS^G12C^ inhibitors, including Sotorasib and Adagrasib, having been approved for use in non‐small‐cell lung cancer or CRC [7]. In addition to these KRAS^G12C^‐specific inhibitors, a range of allele‐specific and pan‐RAS inhibitors/degraders are currently under clinical development [7]. Despite the promising clinical data for RAS inhibitors, the current evidence suggests that resistance development is a significant challenge, which can occur as primary, secondary, or adaptive resistance, thereby limiting the full potential of these therapies [7]. Here, combining RAS inhibitors with drugs that target processes that limit their efficacy is a promising approach [8].

Motivated by a recent prospective multicentered study, which reported a significantly lower overall and clinical complete response to TNT in KRAS mutant RC [9], we investigated our cohort of RC patients. In the retrospective study, we observed a trend towards reduced disease‐free survival and complete pathological responses, particularly in RCs with G12V and G12C mutations. Given that patients with such mutations may require alternative therapies, we investigated the efficacy of RAS inhibitors in RC models and observed rapid adaptive tolerance, which can be overcome by epidermal growth factor receptor (EGFR) family inhibitors.

## 2 Material and Methods

### 2.1 Selection of Patients, Study Design and Treatment

Investigation of rectal cancer tissues and establishment of primary rectal cancer cellular models was approved by the UMG Ethical Committee (Application Nr. 9/8/08). Three hundred ninety patients were included in our retrospective analysis and were treated at the Departments of General, Visceral and Pediatric Surgery at UMG or in 5 cooperating departments throughout Germany. Patients were enrolled in or at least treated according to the CAO/ARO/AIO‐94 [10] or CAO/ARO/AIO‐04 [11] trial of the German Rectal Cancer Study Group (Table [Link], [Link]). Three hundred sixty‐two of the 390 patients were preoperatively treated with RT, 5‐fluorouracil (5‐FU)‐ or 5‐FU and Oxaliplatin‐based CRT followed by surgical resection and pathologic workup standardized according to the guidelines of these randomized phase‐III clinical trials. CRT included a total radiation dose of 50.4 Gy (single dose of 1.8 Gy) accompanied by either 5‐FU (*n* = 197, 50%) or a combination of an intravenous infusion of oxaliplatin and a continuous infusion of 5‐FU (*n* = 127, 33%). 28 patients received no neoadjuvant treatment and 38 patients underwent only radiotherapy (10%). Within 4–6 weeks after completion of preoperative therapy, surgery was performed, including total meso‐rectal excision. Patients with no neo‐adjuvant therapy were excluded from the survival analyses.

### 2.2 Pre‐Therapeutic Tumor Biopsies and Gene‐Expression Microarray Analysis

Biopsies from patients with rectal cancer were taken pre‐therapeutically. mRNA preparation, hybridization to a Human 4 × 44 K v2 gene‐expression Array (Agilent Technologies (G4845A; *n* = 217)) and analysis was recently described [12]. Data can be accessed via the NCBI Expression Omnibus (GSE87211).

### 2.3 KRAS Status and TRG Determination

Primer extension method (SNaPshotTM) was used for initial KRAS mutation status testing for Codons 12, 13, 61, and 146 and discordant results by this method were reevaluated by using the FDA‐approved KRAS Pyro Kit 24, V1 and the RAS Extension Pyro Kit 24, V1 Kit (therascreen1 KRAS test) as described [13, 14]. Tumor regression grade (TRG) of the Goettingen cohort was evaluated using the classification of Dworak et al. [15] and the pathological complete response (pCR) was defined as the absence of viable tumor cells in the primary tumor and lymph nodes (pCR = TRG4). Disease free survival was determined from the start of neoadjuvant therapy. For the MSK cohort, the AJCC/CAP TRG method was used [16], TRGs were taken from [17], and TRG = 0 defines a pCR in this cohort.

### 2.4 Cell Lines

Human rectal cancer cell lines SW837 and SW1463 were acquired from the American Type Culture Collection (Manassas, VA, USA) and cultured in L‐15 Leibovitz medium (#11415049; Gibco, Schwerte, Germany) supplemented with 10% (v/v) fetal calf serum (FCS) (#TMS‐013‐B; Merck Millipore, Berlin, Germany) and 2 mM L‐glutamine (#25030‐081; Gibco, Schwerte, Germany) at 37°C in a CO2‐free incubator. Primary colorectal cancer cell lines GOE‐READ139, GOE‐READ169, and GOE‐READ126 were derived from the patient‐derived organoid (PDO) lines PDO‐PT‐139T, PDO‐PT‐169T, and PDO‐PT‐126T. HROC cell lines were established in University Medical Center Rostock from colorectal cancer patient‐derived xenograft models [18]. For generating 2D‐cell lines, PDOs were mechanically fragmented and seeded in DMEM/F‐12 medium (#D8437; Sigma, Darmstadt, Germany) supplemented with 10% FCS, 2 mM L‐glutamine, and Penicillin/Streptomycin (#15140‐122) in 12‐well plates. The cells were expanded and maintained in this medium. Mutations in primary cell lines were identified using the QIAseq Targeted DNA Custom Panel on an Illumina NextSeq550 Dx at the University Medical Center Goettingen (UMG). HROC111 and HROC147 have their tumor origin in the left colon and HROC402Met1 was derived from a liver metastasis from a primary rectal cancer (Table S2). All cell lines were tested for *mycoplasma* by PCR as described [19].

### 2.5 Patient‐Derived Organoids (PDOs): Generation and Cultivation

PDOs were cultured in media consisting of Advanced DMEM/F‐12 medium (#12634028; Gibco) supplemented with 10 mm HEPES (#15630080; Gibco), 1 x GlutaMAX (#35050061; Gibco), 20% R‐spondin1‐conditioned medium (R‐spondin1‐expressing HEK293T cells), 1 x B27 (#17504001; Thermo‐Fischer), 10 nM Nicotinamide (#N0636; Sigma‐Aldrich), 1.25 mM N‐acetylcysteine (#A9165; Sigma‐Aldrich), 1 x N2 supplement (#17502048 Gibco), 10% Noggin‐conditioned medium (Fc‐tagged mouse Noggin‐expressing HEK293T cells), 50 ng/mL human EGF (Gibco), 1% Penicillin/Streptomycin and 500 nM A83‐01 (#2939; Tocris). 100 μg/mL Primocin (#ant‐pm‐2; Invivogen) was added for extraction and the first passages. After splitting, 10 μM Rho Kinase Inhibitor (Y‐27632) (#Y0503; Sigma‐Aldrich) was added to the medium. For passaging organoids, the matrix was mechanically disrupted in 6 mL ice‐cold PBS. The PDOs were cultured in a 24‐well plate in 50 μL domes of GeltrexTM LDEV‐Free Reduced Growth Factor Basement Membrane Matrix (#A1413202, Gibco). The cell culture plate was incubated in 37°C and 5% CO2 for 30 min for solidification of the matrix before adding the medium.

Extended experimental procedures are available in the Supporting Information S1: Methods section.

## 3 Results

### 3.1 KRAS Mutations and Clinical Outcomes

**FIGURE 1:**
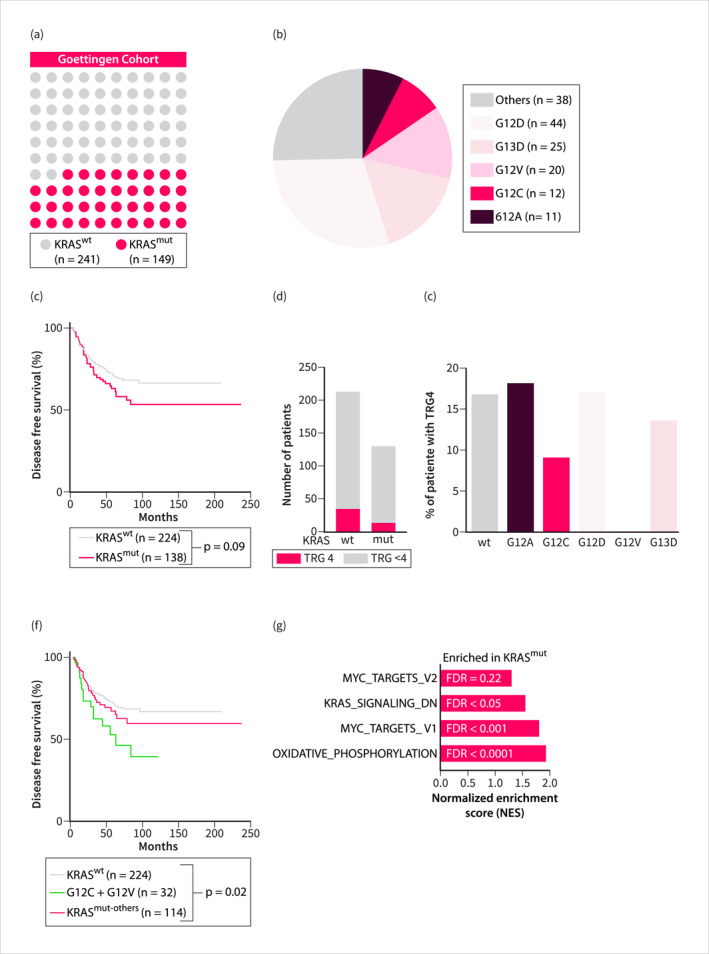
KRAS mutation and clinical outcomes. (a) and (b) Visualization of the distribution of (a) KRAS mutations and (b) codon‐specific KRAS mutations in the Goettingen rectal cancer patient cohort. (c) Kaplan–Meier curve comparing disease‐free survival of KRAS mutation versus WT. (d) Comparison of tumor‐regression grades (TRG) between KRAS wild‐type and mutant RC. TRG4 means complete pathological regression. (e) Percentages of patients with codon‐specific KRAS mutations having had a complete regression (TRG4). (f) Disease‐free survival comparison between RC patients without KRAS mutation, with either a KRAS G12C or G12V mutation and all other KRAS mutations in the Goettingen cohort. (g) GSEA of mRNA microarray data from KRAS mutant (*n* = 84) and KRAS WT patients (*n* = 109). Enriched gene sets are shown for KRAS mutant patients.

To investigate the impact of KRAS mutation on clinical outcomes, we conducted a retrospective analysis of 390 RC patients in the Goettingen RC Cohort (Table S1). Our results showed that the KRAS gene was altered in 38.2% of cases (Figure 1a), with the G12D allele (29.5%) being most frequently observed (Figure 1b). Disease‐free survival (DFS) of neoadjuvant‐treated RC patients with mutated KRAS was reduced (log rank *p*‐value = 0.09) (Figure 1c). The tumor regression grading (TRG) is an established prognostic marker for DFS in patients with locally advanced rectal cancer (LARC) who have undergone neoadjuvant therapy [20]. Our analysis of available TRG scores according to Dworak et al. [15] showed that overall 14.8% of RC patients had an absence of viable tumor cells after neoadjuvant therapy in our retrospective cohort, corresponding to a TRG4 score (Figure S1a). RC patients with a TRG4 had significantly improved DFS (Figure S1b). KRAS mutated RCs have a reduced fraction of TRG4 (11.5%) compared to KRAS wildtype RCs (16.9%; Fisher Exact test, *p* = 0.21) (Figure 1d). Given the functional diversity of KRAS alleles [21], we examined the distribution of the TRG4 fraction among the most frequent alleles. For G12A, G12D, and G13D mutations, the fraction of TRG4 did not differ from that of the wildtype (Figure 1e). In contrast, a TRG4 score was observed in only 9.1% of RCs with the G12C mutation, and in 0% of RCs with the G12V mutation (Fisher Exact test, *p* = 0.0498, not significant after correction for multiple testing; Figure 1e). Consequently, the DFS of neoadjuvant‐treated RC with the G12C or G12V alleles was significantly reduced (Figure 1f). To validate these results, we examined an independent RC cohort that was recently published [17], including patients with varying neoadjuvant treatment regimens. Please note that an alternative system to define tumor regression was used in this study [17]. Across all therapies, no significant association was observed between KRAS mutational status and complete pathological response rates (Figure S1c). However, the combined DFS of patients with G12C or G12V mutations was significantly reduced, highlighting the more aggressive tumor biology associated with these specific variants in an independent cohort (Figure S1d). In our cohort, neoadjuvant therapy included radiotherapy alone, 5‐FU‐based CRT, or 5‐FU plus Oxaliplatin‐based CRT (Figure S2a, Table S1). In patients with KRAS G12C or G12V mutations, the proportion receiving either radiotherapy alone or 5‐FU plus oxaliplatin‐based CRT was higher (Figure S2a). However, the reduced DFS in patients with KRAS G12C or G12V mutations persisted even among those receiving more aggressive treatment with 5‐FU plus Oxaliplatin‐based CRT (Figure S2b). In the validation cohort, treatments with neo‐adjuvant CRT, consolidation, or induction regimens were included (Figure S2c). Mutated KRAS was associated with shorter DFS in patients treated with CRT followed by consolidative chemotherapy [17]. Notably, the impact of KRAS mutant alleles on DFS was primarily associated with the G12C and G12V variants (Figure S2d). High inter‐patient heterogeneity restricted differential expression to 131 genes in KRAS‐mutant RC, with gene set enrichment (GSEA) analysis showing only a few HALLMARK pathways enriched (Figure 1g). These include MYC and oxidative phosphorylation signatures, which provide a link between RAS, metabolism, and MYC in RC. In summary, KRAS mutated RC has reduced DFS and certain alleles, such as the G12V and G12C alleles, show reduced responsiveness to neoadjuvant therapies.

### 3.2 Adaptive Response of RC Cells to KRAS Inhibitors

**FIGURE 2:**
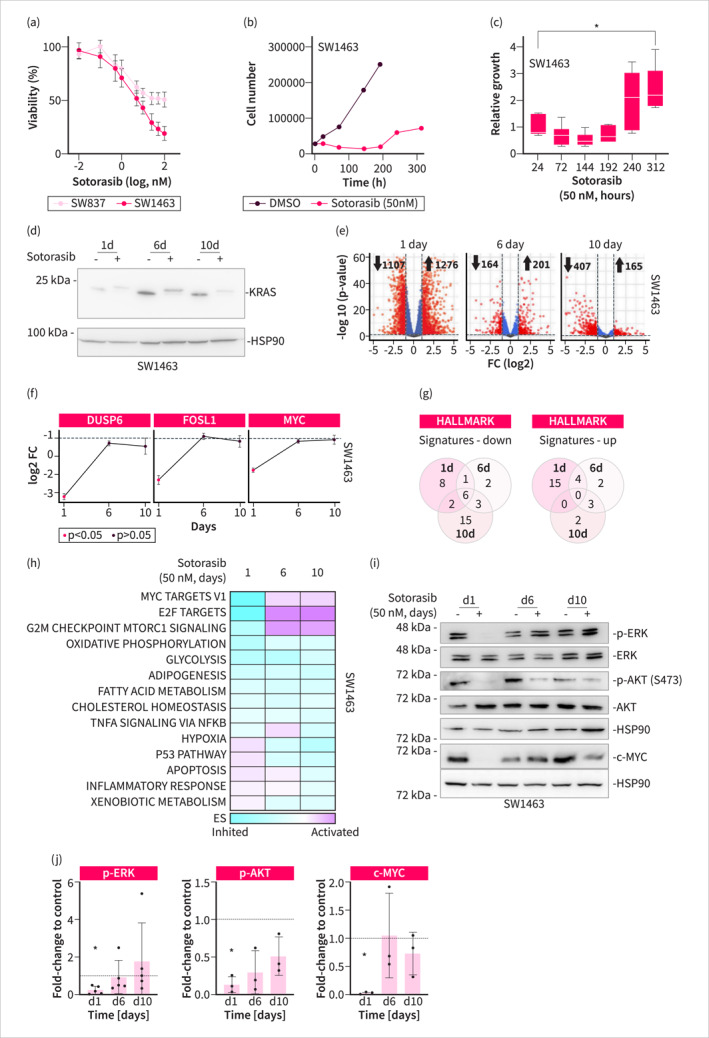
Rapid adaptation of SW1463 cells to sotorasib treatment. (a) Sotorasib dose‐response curve measured by ATP‐dependent CellTiter Glo reagent after a treatment period of 3 days. (b) Cell number was counted over 13 days upon Sotorasib treatment. *n* = 3. (c) Relative growth was calculated based on data from (b). *ANOVA *p* < 0.05. (d) Demonstration of KRAS inhibition by covalent binding of Sotorasib to KRAS, inducing a mobility shift in Western blots. *n* = 3. (e) Volcano plots derived from RNA‐sequencing data of SW1463 cells treated for 1, 6, and 10 days. The number of downregulated (left) and upregulated (right) genes is indicated (treated vs. DMSO control). (f) Binary logarithmic fold‐change of specific KRAS downstream targets. (g) Venn diagram of Hallmark gene sets with a *q*‐value < 0.05 computed by a GSEA from Sotorasib‐treated SW1463 cells. Left panel: signatures depleted in Sotorasib‐treated SW1463 cells, Right panel: signatures enriched in Sotorasib‐treated SW1463 cells. (h) Color‐coded enrichment scores of Hallmark gene sets in GSEA over time. All shown signatures have a *q*‐value < 0.05. (i) Western blot analysis of SW1463 cells that were treated with 50 nM Sotorasib over 10 days. HSP90 serves as a loading control. (j) Western blot was quantified by intensity measurement and ratios were calculated to DMSO control, which were arbitrary set to 1. p‐ERK/pan‐ERK *n* = 5; p‐AKT/pan‐AKT *n* = 3; MYC *n* = 3. Significance was tested using Student's t‐test on normalized protein density and * indicates a *p* value < 0.05.

Given the observation that KRAS mutant RCs have a reduced DFS and the recent development of direct RAS inhibitors, we explored these inhibitors in RC cells. Considering the advanced development stage of Sotorasib [7], we investigated the response in the KRAS^G12C^ mutant RC cell lines SW837 and SW1463. SW1463 cells exhibited higher sensitivity to Sotorasib compared to SW837 cells, with a calculated half‐maximal growth inhibitory concentration (GI_50_) of 6 nM (Figure 2a). Despite the initial sensitivity of SW1463 cells to Sotorasib, we observed a rapid adaptation of the cells to the treatment even to a high dose of 50 nM Sotorasib, as evidenced by the restart of cellular growth after 6 days of continuous treatment (Figure 2b,c). This was further confirmed by re‐seeding SW1463 cells after 6 days of continuous treatment. Here, a right shift in the Sotorasib dose‐response curve was observed, showing adaptation (Figure S3a). Long‐term treatment with gradually increasing Sotorasib doses over 6 weeks (Figure S3b) demonstrated that SW1463 cells exhibit minimal further adaptation beyond the initial 2 weeks (Figure S3a,c), supporting the note that adaptation occurs early upon KRAS inhibition. We monitored KRAS engagement by the inhibitor by assessing the electrophoretic mobility of KRAS in Western blots. Sotorasib binds covalently to KRAS and we observed reduced electrophoretic mobility during the treatment period of 10 days (Figure 2d). To monitor the adaptive response to Sotorasib at the level of the transcriptome, we conducted RNA sequencing analysis. In addition, we observed an adaptive response of perturbed transcripts upon the treatment with Sotorasib. While more than 1000 genes were up or downregulated 24 h after treatment with Sotorasib, the number decreased to a few hundred after 6 and 10 days of treatment, indicating that the oncogenic transcriptome is at least partially restored (Figure 2e). This is also evident at the level of single transcripts. Target genes of the canonical KRAS pathway, such as FOSL1 and DUSP6, or prominent oncogenes, such as MYC, were significantly inhibited after 24 h of Sotorasib treatment, but returned to baseline expression after 6 and 10 days of treatment (Figure 2f, Table S3). Additionally, gene set enrichment analysis (GSEA) revealed significant temporal regulation of HALLMARK signatures, indicating pathways that were either activated or suppressed in response to Sotorasib (Figure 2g). Based on the temporal regulation of these signatures, we identified three distinct functional groups. The growth phenotype (Figure 2b,c) is mirrored by the regulation of MYC, E2F, and G2M checkpoint HALLMARK signatures. While these signatures are significantly inhibited after 24 h of treatment with Sotorasib, they are even more enriched in Sotorasib‐treated cells after 6 and 10 days (Figure 2h). Furthermore, we identified two additional groups. Metabolic signatures, including oxidative phosphorylation, glycolysis, and lipid metabolism, remained inhibited throughout the entire period (Figure 2h). Lastly, a group of stress‐indicating signatures, including p53 and apoptosis signatures, as well as inflammatory signatures, were up‐regulated early upon KRAS inhibition but returned to basal levels later (Figure 2h). At the protein level, phosphorylation of ERK and AKT, as well as c‐MYC expression, is distinctly decreased 24 h after Sotorasib treatment but was partially or completely restored at later time points (Figure 2i,j). These data highlight the limitations of RAS inhibitors in RC. While SW837 cells are rather resistant to the investigated inhibitor, SW1463 cells quickly adapt and grow under KRAS blockade.

### 3.3 Regulation of the EGF Family Ligands and Receptors by Sotorasib

To identify a RAS inhibitor combination therapy in the context of RC with the goal of enhancing efficacy, we employed a drug screening approach using a Sotorasib‐anchored drug screening design (Figure 3a, Table S4), which has been successfully applied in our previous studies [19, 22]. Using a delta of the area under the dose‐response curve (AUC) of mono‐ and combination therapies of less than −0.1 as a criterion for a hit, we identified 22 hits in SW1463 (Figure 3b) and SW837 (Figure 3c) cells each (Table S4). A Venn analysis demonstrated an overlap of eight common hits in both RC cell lines (Figure 3d). These hits include Linsitinib, an IGF‐1R inhibitor; Neratinib, an ERBB2 and EGFR receptor inhibitor; Binimetinib, a MEK inhibitor; GSK3368715, a PRMT1 inhibitor; SHP099, a SHP2 inhibitor; Tipifarnib, a farnesyltransferase inhibitor; BI‐3406, a SOS inhibitor; and NVP‐CGM097, a MDM2 inhibitor. A STRING analysis of the targets revealed a tight connection between them, particularly for the receptor tyrosine kinases (RTKs) and their associated downstream signaling pathways (Figure 3f). These data suggest that a common pathway, signaling via RTKs and canonical MEK‐ERK pathways, is involved in adaptation and restores the oncogenic transcriptome. Interestingly, we observed the induction of various EGFR signatures induced after 24 h of Sotorasib treatment (Figure 3g), which was connected to the up‐regulation of EGFR ligands and especially the receptors ERBB2 and ERBB3 (Figure 3h,iI). On the other hand, the gene expression of EGFR was decreased. The up‐regulation of ERBB2 and ERBB3 upon Sotorasib treatment was further confirmed by Western blotting (Figure 3j). Moreover, we observed downregulation of EGFR following 24 h of Sotorasib treatment, with levels recovering after 6 days (Figure 3k). Phosphorylation of the EGFR was increased at the investigated time points (Figure 3k). Integration of Sotorasib‐responsive genes with ERBB2/ERBB3 transcription factor prediction databases revealed candidate transcriptional regulators (Figure S4). To determine whether long‐term adaptation of SW1463 cells alters their pharmacological vulnerabilities, we conducted a drug screening assay in cells adapted to Sotorasib over 6 weeks (Figure S3b). Although EGFR signatures normalize after several days of treatment, similar to most gene sets, EGFR family inhibitors remain upon the top scoring hits (Table S4 and Figure S3d). The combination of Sotorasib and Neratinib remained synergistic even in Sotorasib‐adapted cells (Figure S3e,f). Given the strong connection to EGFR signaling, we chose to further validate the combination of RAS and EGFR inhibition.

FIGURE 3Receptor‐tyrosine kinase‐mediated adaptation identified by drug‐screening. (a) Scheme showing unbiased drug‐combination screening of SW1463 and SW837 cells with an ATP‐based read‐out. (b) and (c) Distribution of the delta AUC of the Sotorasib‐anchored drug screening experiment (50 nM Sotorasib) in (b) SW1463 and (c) SW837 cells. (d) Venn diagram of synergistic screening hits with delta AUC < −0.1 shows overlap between both cell lines. (e) Heatmap of AUC values of the eight overlapping drug combinations. Corresponding to (c). (f) STRING analysis of drug targets from overlapping screening hits. (g) Upregulated ERBB‐family‐related gene sets from multiple databases after 24 h of Sotorasib treatment. (h) Heatmap of ERBB family and selected ligands based on mRNA expression values after 24 h of treatment with Sotorasib (50 nM) in SW1436 cells. (i) Expression of selected EGFR family ligands and receptors over time. (j) Protein expression of ERBB2 and ERBB3 in Western blot. Left: Representative blot of three independent replicates. Right: Quantification. Significance was tested by Student's t‐test on normalized protein density and * indicates a *p* value < 0.05 or the *p* value is indicated. (k) Western blot of EGFR and Tyr1068 phosphorylated EGFR. Left panel: one representative experiment. Right panel: Quantification of three independent experiments. *p value < 0.05 determined as in (j).

**Figure:**
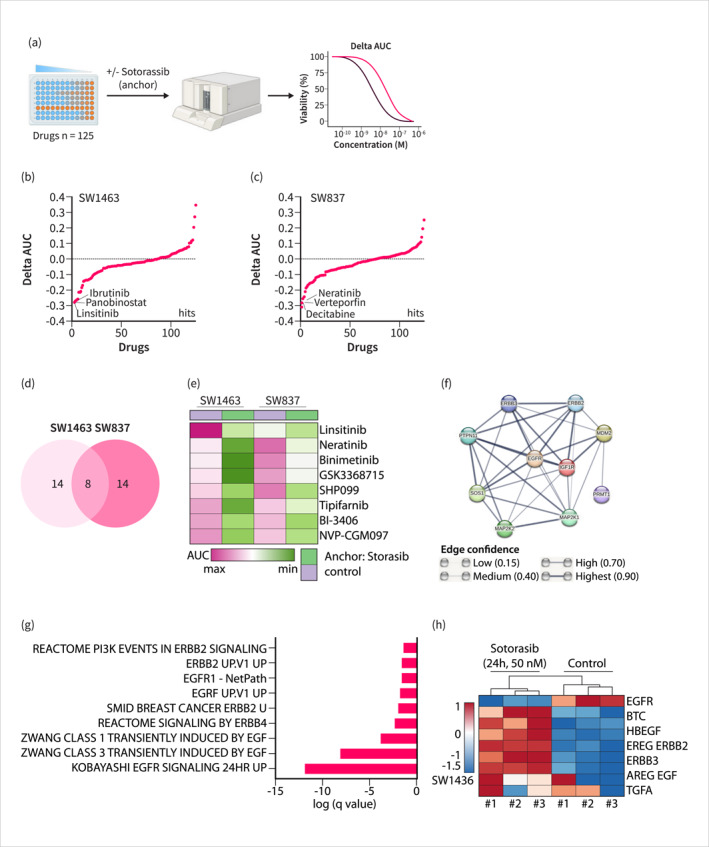

**Figure:**
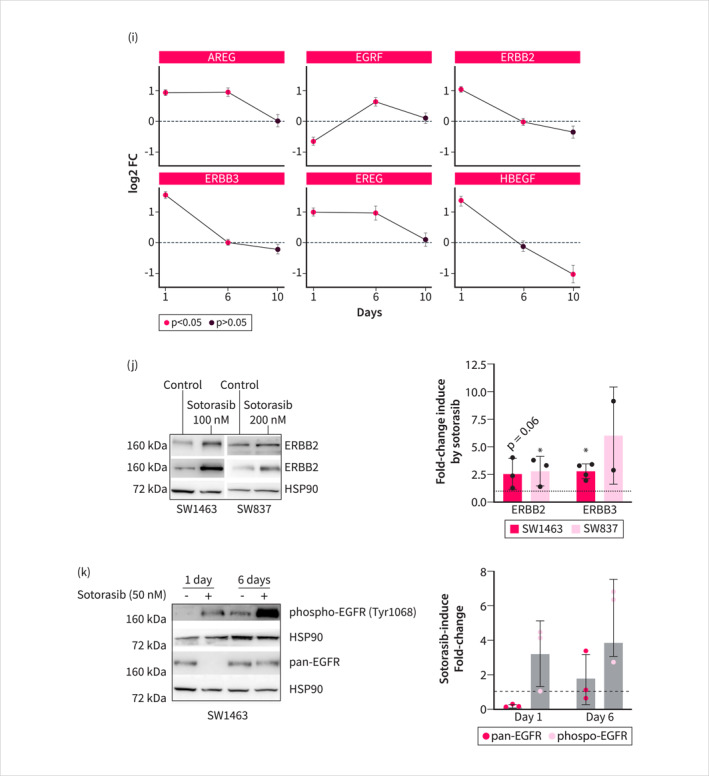

### 3.4 The RAS and EGFR Inhibitor Combination Induces Apoptosis

To elucidate the underlying mechanism contributing to the observed synergism between RAS and EGFR family inhibitors, we investigated associated signaling pathways. Our analysis revealed an impact of the combination therapy on the phosphorylation of ERK and AKT (Figure 4a), suggesting that EGFR family signaling plays a role in maintaining these pathways despite covalent KRAS inhibition. We analyzed the proteomes of SW1463 cells treated with Sotorasib alone or in combination with Neratinib for 24 h (Table S5). Using GSEA analysis of differentially expressed proteins, we detected the downregulation of pro‐proliferative signatures—including MYC‐, E2F‐, and G2M‐associated pathways—in cells treated with the Sotorasib‐Neratinib combination. These signatures encompass cell cycle related proteins such as AURKB or MCM5 (Figure 4b,c). Furthermore, the Sotorasib‐Neratinib combination therapy upregulated apoptosis‐related signatures, including the pro‐apoptotic protein BIM (BCL2L11; Figure 4b,c). Consistent with the apoptosis signature, we detected an increased cleaved 24 kDa PARP fragment levels in SW837 cells (Figure 4d). In summary, the combination of RAS and EGFR inhibitors effectively blocks RC relevant pro‐oncogenic pathways and induces apoptosis.

FIGURE 4Validation of the KRASi and EGFRi combination therapy. (a) Sotorasib and Neratinib combinatorial inhibition of ERK and AKT activation in Western blot. (b) Mass Spectrometry measurement of SW1463 cell lysate treated with 20 nM Sotorasib alone or in combination with 20 nM Neratinib (*n* = 3). Gene names of measured peptides are shown. Red dots: cell cycle regulators, blue dots: apoptosis regulators. (c) GSEA on proteome data. All Hallmark gene sets with FDR < 0.1 are shown. (d) Western blot of the cleaved 24 kDa PARP fragment upon combination treatment. All cells, including detached ones, were lysed and analyzed by Western blot. (e) PDO treatment with pan‐KRAS inhibitor BI‐2865 and subsequent brightfield imaging. Red Arrow: PDO Blebbing. (f) Bliss synergy scores calculated with the Synergy finder from ATP‐based Cell Titer Glo viability measurements. (g) Oncoprint showing genomic alterations in cell lines annotated with their primary tumor sites of origin. (h) Maximum Bliss synergy scores were observed for the pan‐RAS inhibitor RMC‐6236 in combination with Neratinib, based on CellTiter‐Glo ATP assays (a surrogate for cell viability). A 7 × 5 dose‐response matrix with 5‐fold dilution of RMC‐6236 and 4‐fold dilution of Neratinib was applied (*n* ≥ 2). (i) Clonogenic assay of GOE‐READ169. (j) Scheme of in situ resistance assay with weekly treatments over the duration of the assay. (k) Kaplan–Meier curve of in situ resistance assay with GOE‐READ139. All Western blots and clonogenic assays are representative images out of *n* = 3 biological replicates.

**Figure:**
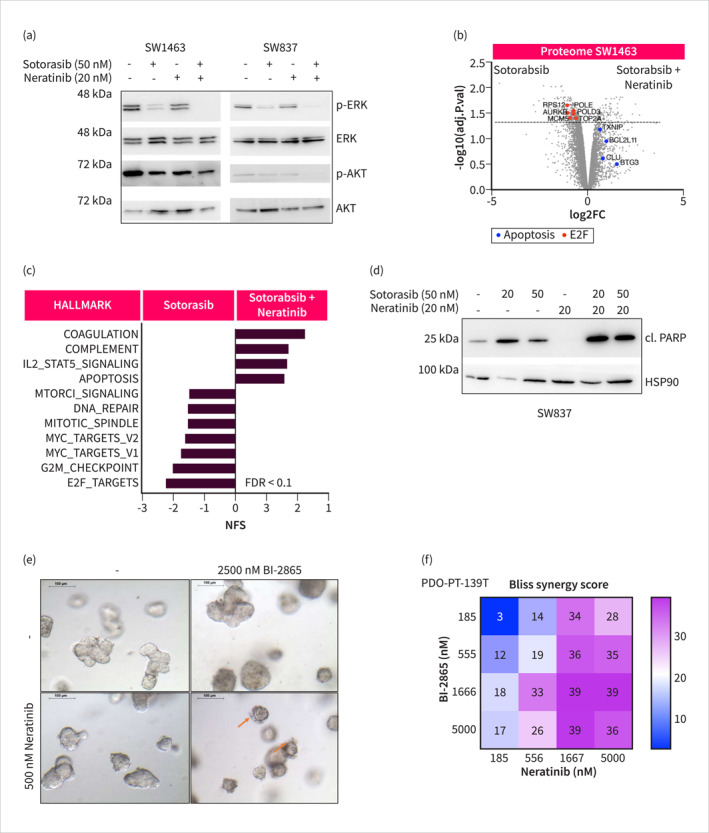

**Figure:**
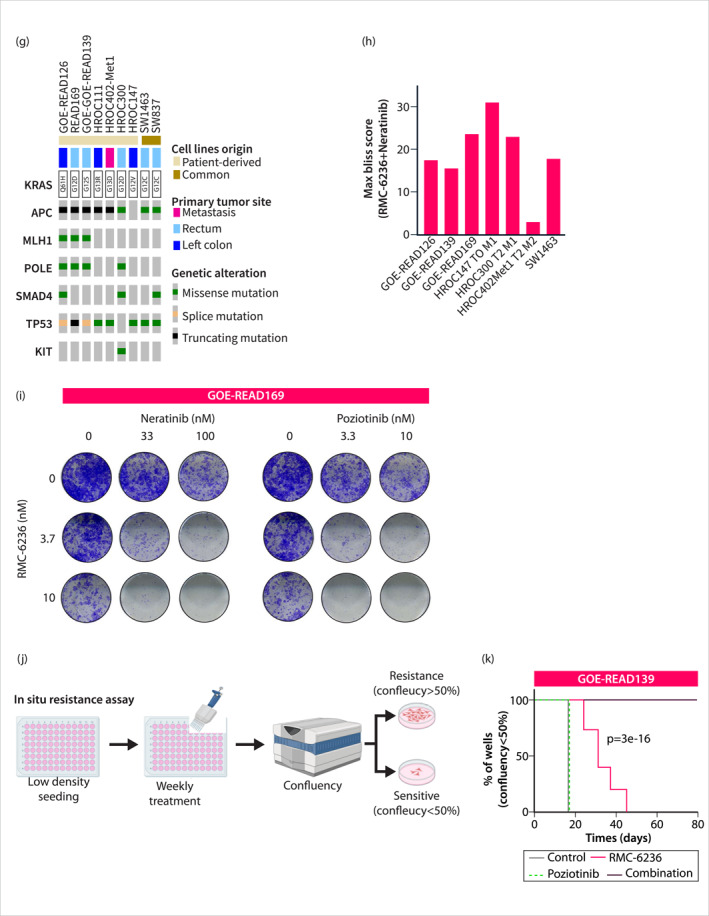

### 3.5 The KRASi Combination With EGFR Family Inhibitors in Primary Patient‐Derived Models

To further validate the efficacy of combining KRASi with EGFR family inhibitors, we utilized KRAS‐mutated primary patient‐derived organoids (PDOs) and patient‐derived cell lines (PDCLs). In the RC PDO model PDO‐PT139T, we observed significant synergistic effects between the pan‐KRAS inhibitor BI‐2865 [23] and Neratinib (Figure 4e,f). We interpret organoid blebbing (arrowheads in microscopy images of combinatorial‐treated lines) as an additional indicator of cell death. Similarly, in KRAS‐mutated PDCLs (Figure 4g and Table S2), the combination of the RAS MULTI (ON) inhibitor RMC‐6236 [24, 25] with Neratinib demonstrated superior efficacy compared to either agent alone, resulting in Bliss synergy scores above 10 in six out of seven tested models, indicating synergistic activity (Figure 4h). Furthermore, clonogenic growth was distinctly impaired in combinations of RMC‐6236 and Neratinib or Poziotinib co‐treated cells (Figure 4i). To further explore the potential for long‐term tumor growth suppression, we employed the in situ resistance assay (ISRA), a model designed to simulate acquired resistance to targeted therapies [26] (Figure 4j). Poziotinib demonstrated no therapeutic efficacy as a monotherapy in this assay (Figure 4k). Consistent with the rapid adaptation to KRAS inhibition observed, we detected complete outgrowth of the RMC‐6236‐treated PDCL model GOE‐READ139 (Figure 4k). In contrast, the addition of the EGFR family inhibitor Poziotinib to RMC‐6236 completely prevented tumor outgrowth over the 80‐day investigation period, highlighting the significantly enhanced efficacy of the combination therapy (Figure 4k). In summary, we demonstrated that the synergistic effect of co‐targeting RAS and EGFR family members is consistent across diverse cell lines, inhibitors, and treatment durations.

## 4 Discussion

We identified KRAS gene alterations, predominantly at codon 12, in approximately 40% of RC cases in our cohort and a trend towards reduced DFS in KRAS mutated cases. A recent prospective multicenter clinical trial demonstrated a significantly lower overall and clinical complete response rate in the “mutRAS” group following TNT [9]. Although the “mutRAS” group included a few NRAS and BRAF mutant RCs [9], the study highlights the relevance of KRAS mutations as a response predictive biomarker and suggests considering alternative therapies to improve the rate of complete responses. This note is also underscored by retrospective studies or meta‐analysis reporting an impact of the KRAS mutational status on DFS [17] or complete responses to neoadjuvant therapy [27, 28, 29]. We observed a reduced fraction of tumors with pathologic complete responses according to Dworak et al. [15] in RC patients with G12V and G12C KRAS mutations, which was associated with a significant reduction in DFS when combining both alleles. While specific impacts of KRAS alleles, such as CRT resistance for codon 13 mutant KRAS alleles, have been reported [28, 30], their value needs to be investigated in prospective clinical trials and the underlying molecular processes deciphered in further experimental studies. Because of a worse DFS and reduced CRT response, especially RC patients with KRAS mutations could benefit from KRAS inhibitors if resistance challenges can be overcome.

To address this, we investigated the relevance of KRASi in KRAS^G12C^‐mutated pre‐clinical RC models. While SW837 cells were relatively resistant to Sotorasib, we observed a rapid adaptive tolerance in SW1463 cells. This critical adaptation occurred primarily within the first week, followed by a significantly slower increase in tolerance development, underscoring the importance of rapid cellular rewiring processes. Adaptation, an important process for limiting KRASi efficacy [7], operate across multiple layers: at the cellular level, through the restoration of proliferation; at the signaling level, with restoration of canonical and non‐canonical RAS signaling, at the transcriptome level, marked by significant deregulation after 1 day followed by gradual normalization over time; at the oncogenic driver expression level, with the re‐emergence of key oncogenes such as FOSL1 and MYC; and at the pathway level, characterized by an initial suppression of pro‐proliferative, and MYC‐driven signatures, which are subsequently restored during later adaptive stages. Although the relevance of FOSL1 and MYC in limiting the efficacy of inhibitors of the canonical RAS pathway and RASi is documented [25, 31, 32, 33, 34, 35], their functional impact on adaptation needs to be clarified in future experiments. Furthermore, the transcription and epigenetic factors potentially responsible for restoring the oncogenic program remain unclear and require further investigation.

The limited efficacy of the KRASG12C inhibitors in RC prompted us to perform an unbiased drug screening experiment. We detected screening hits centered around RTKs and their associated down‐stream signaling. In metastatic CRC, the combination of KRAS inhibitors with the EGFR antibodies Panitumumab and Cetuximab are approved by the FDA [36, 37]. This is based on strong pre‐clinical evidence that places the EGFR receptor and signaling to KRASi resistance and upstream of restoring the activity of the canonical RAS‐RAF‐MEK‐ERK activity, that might involve activation of wild‐type RAS [38, 39, 40, 41, 42]. Our findings demonstrate that KRAS inhibitor adaptation is driven by rapid transcriptomic upregulation of EGFR signaling components, including the receptors ERBB2 and ERBB3 as well as ligands. Although Sotorasib treatment initially reduces EGFR mRNA and protein expression, we observed increased phosphorylation, suggesting potential heterodimerization with receptors like ERBB2 or ERBB3. However, this possibility requires further experimental validation.

Long‐term Sotorasib treatment studies indicate persistent dependence on EGFR‐family‐mediated signaling. The irreversible EGFR/ERBB2 inhibitor Neratinib, which is approved for the treatment of ERBB2‐positive breast cancers [43], emerged as a hit in our drug screening experiment for KRASi‐based combination therapies. Consistently, the combination of the irreversible pan‐ERBB inhibitor Poziotinib and the RAS MULTI (ON) inhibitor RMC‐6236 demonstrated a remarkable ability to prevent the long‐term outgrowth of primary patient‐derived RC models, highlighting a potential therapeutic option of small‐molecule inhibitors targeting EGFR family receptors as an alternate strategy to Panitumumab and Cetuximab.

In summary, our findings further point to the negative impact of KRAS mutations, particularly the G12C and G12V alleles, on the response to neoadjuvant therapies. Additionally, the results show the potential of incorporating small‐molecule pan‐ERBB inhibitors into KRAS inhibitor‐based combination treatments.

## Author Contributions

All data and the work reported in the paper have been obtained or were generated by the authors, unless clearly specified in the text. Conception and design of the study: J.B., M.S., H.Z., C.T.C., M.Gr., J.G., G.S. Acquisition of data and/or analysis, curation, and interpretation of data: J.B., M.S., X.F., H.Z., C.T.C., C.S., S.K., F.R., M.W., J.G., G.S. Generation of important models and contribution of essential resources, technology, and funding: J.B., M.S., H.Z., T.dO., M.L., F.R., L.C., M.Gh., M.Gr., J.G., G.S. Drafting of the manuscript: J.B., J.G., G.S. Revision for important intellectual content: all authors. Approval of the final version for publication: all authors.

## Ethics Statement

The study was approved by the local ethics committees (number 9/8/08).

## Consent

Written informed consent from the patients for research use was obtained prior to the investigation.

## Conflicts of Interest

The authors declare no conflicts of interest.

## Supporting information

Supporting Information S1

Supporting Information S2

Figure S1

Figure S2

Figure S3

Figure S4

Table S1

Table S2

Table S3

Table S4

Table S5

## Data Availability

Uncropped scans and source data are available upon request. The RNA‐seq and proteomics data of Sotorasib treated SW1463 cells can be accessed via Tables S2 and S4. Previously published microarray data of the RC cohort can be accessed via GSE87211.

## References

[1] I. Ben‐Aharon , H. W. M. van Laarhoven , E. Fontana , R. Obermannova , M. Nilsson , and F. Lordick , “Early‐Onset Cancer in the Gastrointestinal Tract Is on the Rise—Evidence and Implications,” Cancer Discovery 13, no. 3 (2023): 538–551, 10.1158/2159-8290.cd-22-1038. 36757194

[2] M. Ghadimi , C. Rödel , R. Hofheinz , H. Flebbe , and M. Grade , “Multimodal Treatment of Rectal Cancer,” Deutsches Ärzteblatt International 119, no. 33–34 (2022): 570–580, 10.3238/arztebl.m2022.0254. 35791271 PMC9743213

[3] H. Williams , E. Fokas , M. Diefenhardt , et al., “Survival Among Patients Treated With Total Mesorectal Excision or Selective Watch‐and‐Wait After Total Neoadjuvant Therapy: A Pooled Analysis of the CAO/ARO/AIO‐12 and OPRA Randomized Phase II Trials,” Annals of Oncology 36, no. 5 (2025): 543–547, 10.1016/j.annonc.2025.01.006. 39848335 PMC12034476

[4] Y. Kagawa , J. J. Smith , E. Fokas , et al., “Future Direction of Total Neoadjuvant Therapy for Locally Advanced Rectal Cancer,” Nature Reviews Gastroenterology & Hepatology 21, no. 6 (2024): 444–455, 10.1038/s41575-024-00900-9. 38485756 PMC11588332

[5] I. A. Prior , F. E. Hood , and J. L. Hartley , “The Frequency of Ras Mutations in Cancer,” Cancer Research 80, no. 14 (2020): 2969–2974, 10.1158/0008-5472.can-19-3682. 32209560 PMC7367715

[6] M. H. Hofmann , D. Gerlach , S. Misale , M. Petronczki , and N. Kraut , “Expanding the Reach of Precision Oncology by Drugging all KRAS Mutants,” Cancer Discovery 12, no. 4 (2022): 924–937, 10.1158/2159-8290.cd-21-1331. 35046095 PMC9394389

[7] T. Isermann , C. Sers , C. J. Der , and B. Papke , “KRAS Inhibitors: Resistance Drivers and Combinatorial Strategies,” Trends in Cancer 11, no. 2 (2025): 91–116, 10.1016/j.trecan.2024.11.009. 39732595 PMC13308734

[8] H. Jin , L. Wang , and R. Bernards , “Rational Combinations of Targeted Cancer Therapies: Background, Advances and Challenges,” Nature Reviews Drug Discovery 22, no. 3 (2023): 213–234, 10.1038/s41573-022-00615-z. 36509911

[9] S. Bedrikovetski , L. Traeger , T. Fitzsimmons , et al., “Association Between RAS/BRAF Mutations and Complete Response Following Total Neoadjuvant Therapy in Patients With Rectal Cancer: A Prospective Multicentered Study,” Annals of Surgical Oncology 31, no. 3 (2024): 1681–1689, 10.1245/s10434-023-14722-7. 38071720

[10] R. Sauer , T. Liersch , S. Merkel , et al., “Preoperative Versus Postoperative Chemoradiotherapy for Locally Advanced Rectal Cancer: Results of the German CAO/ARO/AIO‐94 Randomized Phase III Trial After a Median Follow‐Up of 11 Years,” Journal of Clinical Oncology 30, no. 16 (2012): 1926–1933, 10.1200/jco.2011.40.1836. 22529255

[11] C. Rödel , T. Liersch , H. Becker , et al., “Preoperative Chemoradiotherapy and Postoperative Chemotherapy With Fluorouracil and Oxaliplatin Versus Fluorouracil Alone in Locally Advanced Rectal Cancer: Initial Results of the German CAO/ARO/AIO‐04 Randomised Phase 3 Trial,” Lancet Oncology 13, no. 7 (2012): 679–687, 10.1016/s1470-2045(12)70187-0. 22627104

[12] G. Emons , N. Auslander , P. Jo , et al., “Gene‐Expression Profiles of Pretreatment Biopsies Predict Complete Response of Rectal Cancer Patients to Preoperative Chemoradiotherapy,” British Journal of Cancer 127, no. 4 (2022): 766–775, 10.1038/s41416-022-01842-2. 35597871 PMC9381580

[13] P. Jo , A. König , M. Schirmer , et al., “Heterogeneity of KRAS Mutation Status in Rectal Cancer,” PLoS One 11, no. 4 (2016): e0153278, 10.1371/journal.pone.0153278. 27064574 PMC4827807

[14] P. Jo , M. Bernhardt , M. Nietert , et al., “KRAS Mutation Status Concordance Between the Primary Tumor and the Corresponding Metastasis in Patients With Rectal Cancer,” PLoS One 15, no. 10 (2020): e0239806, 10.1371/journal.pone.0239806. 33002027 PMC7529221

[15] O. Dworak , L. Keilholz , and A. Hoffmann , “Pathological Features of Rectal Cancer After Preoperative Radiochemotherapy,” International Journal of Colorectal Disease 12, no. 1 (1997): 19–23, 10.1007/s003840050072. 9112145

[16] A. G. Mace , R. K. Pai , L. Stocchi , and M. F. Kalady , “American Joint Committee on Cancer and College of American Pathologists Regression Grade,” Diseases of the Colon & Rectum 58, no. 1 (2015): 32–44, 10.1097/dcr.0000000000000266. 25489692

[17] W. K. Chatila , J. K. Kim , H. Walch , et al., “Genomic and Transcriptomic Determinants of Response to Neoadjuvant Therapy in Rectal Cancer,” Nature Medicine 28, no. 8 (2022): 1646–1655, 10.1038/s41591-022-01930-z. PMC9801308 35970919

[18] C. S. Mullins , B. Micheel , S. Matschos , et al., “Integrated Biobanking and Tumor Model Establishment of Human Colorectal Carcinoma Provides Excellent Tools for Preclinical Research,” Cancers 11, no. 10 (2019): 1520, 10.3390/cancers11101520. 31601052 PMC6826890

[19] C. T. Contreras , J. D. Falke , D. Seifert , et al., “KRASG12C‐Inhibitor‐Based Combination Therapies for Pancreatic Cancer: Insights From Drug Screening,” Molecular Oncology 19, no. 2 (2025): 295–310, 10.1002/1878-0261.13725. 39253995 PMC11792994

[20] C. Rödel , P. Martus , T. Papadoupolos , et al., “Prognostic Significance of Tumor Regression After Preoperative Chemoradiotherapy for Rectal Cancer,” Journal of Clinical Oncology 23, no. 34 (2005): 8688–8696, 10.1200/jco.2005.02.1329. 16246976

[21] R. A. Burge and G. A. Hobbs , “Not all RAS Mutations Are Equal: A Detailed Review of the Functional Diversity of RAS Hot Spot Mutations,” Advances in Cancer Research 153 (2021): 29–61, 10.1016/bs.acr.2021.07.004. 35101234

[22] C. Falcomatà , S. Bärthel , S. A. Widholz , et al., “Selective Multi‐Kinase Inhibition Sensitizes Mesenchymal Pancreatic Cancer to Immune Checkpoint Blockade by Remodeling the Tumor Microenvironment,” Nature Cancer 3 (2022): 318–336, 10.1038/s43018-021-00326-1. 35122074 PMC7612546

[23] D. Kim , L. Herdeis , D. Rudolph , et al., “Pan‐KRAS Inhibitor Disables Oncogenic Signalling and Tumour Growth,” Nature 619, no. 7968 (2023): 160–166, 10.1038/s41586-023-06123-3. 37258666 PMC10322706

[24] M. Holderfield , B. J. Lee , J. Jiang , et al., “Concurrent Inhibition of Oncogenic and Wild‐Type RAS‐GTP for Cancer Therapy,” Nature 629, no. 8013 (2024): 919–926, 10.1038/s41586-024-07205-6. 38589574 PMC11111408

[25] J. Jiang , L. Jiang , B. J. Maldonato , et al., “Translational and Therapeutic Evaluation of RAS‐GTP Inhibition by RMC‐6236 in RAS‐Driven Cancers,” Cancer Discovery 14, no. 6 (2024): 994–1017, 10.1158/2159-8290.cd-24-0027. 38593348 PMC11149917

[26] N. E. Sealover , P. T. Theard , J. M. Hughes , A. J. Linke , B. R. Daley , and R. L. Kortum , “In Situ Modeling of Acquired Resistance to RTK/RAS‐Pathway‐Targeted Therapies,” iScience 27, no. 1 (2024): 108711, 10.1016/j.isci.2023.108711. 38226159 PMC10788224

[27] J. Gaedcke , M. Grade , K. Jung , et al., “KRAS and BRAF Mutations in Patients With Rectal Cancer Treated With Preoperative Chemoradiotherapy,” Radiotherapy & Oncology 94, no. 1 (2010): 76–81, 10.1016/j.radonc.2009.10.001. 19913317 PMC7373270

[28] K. Hammarström , L. Nunes , L. Mathot , et al., “Clinical and Genetic Factors Associated With Tumor Response to Neoadjuvant (Chemo)Radiotherapy, Survival and Recurrence Risk in Rectal Cancer,” International Journal of Cancer 155, no. 1 (2024): 40–53, 10.1002/ijc.34880. 38376070

[29] E. D. Mattia , J. Polesel , S. Mezzalira , et al., “Predictive and Prognostic Value of Oncogene Mutations and Microsatellite Instability in Locally‐Advanced Rectal Cancer Treated With Neoadjuvant Radiation‐Based Therapy: A Systematic Review and Meta‐Analysis,” Cancers 15, no. 5 (2023): 1469, 10.3390/cancers15051469. 36900260 PMC10001009

[30] O. S. Chow , D. Kuk , M. Keskin , et al., “KRAS and Combined KRAS/TP53 Mutations in Locally Advanced Rectal Cancer Are Independently Associated With Decreased Response to Neoadjuvant Therapy,” Annals of Surgical Oncology 23, no. 8 (2016): 2548–2555, 10.1245/s10434-016-5205-4. 27020587 PMC5047012

[31] C. Schneeweis , S. Diersch , Z. Hassan , et al., “AP1/Fra1 Confers Resistance to MAPK Cascade Inhibition in Pancreatic Cancer,” Cellular and Molecular Life Sciences 80, no. 1 (2023): 12, 10.1007/s00018-022-04638-y. PMC9763154 36534167

[32] U. N. Wasko , J. Jiang , T. C. Dalton , et al., “Tumor‐Selective Activity of RAS‐GTP Inhibition in Pancreatic Cancer,” Nature 629, no. 8013 (2024): 927–936, 10.1038/s41586-024-07379-z. 38588697 PMC11111406

[33] R. Sulahian , J. J. Kwon , K. H. Walsh , et al., “Synthetic Lethal Interaction of SHOC2 Depletion With MEK Inhibition in RAS‐Driven Cancers,” Cell Reports 29, no. 1 (2019): 118–134.e8, 10.1016/j.celrep.2019.08.090. 31577942 PMC6918830

[34] K. Lou , V. Steri , A. Y. Ge , et al., “KRASG12C Inhibition Produces a Driver‐Limited State Revealing Collateral Dependencies,” Science Signaling 12, no. 583 (2019): eaaw9450, 10.1126/scisignal.aaw9450. 31138768 PMC6871662

[35] J. Dilly , M. T. Hoffman , L. Abbassi , et al., “Mechanisms of Resistance to Oncogenic KRAS Inhibition in Pancreatic Cancer,” Cancer Discovery 14, no. 11 (2024): 2135–2161, 10.1158/2159-8290.cd-24-0177. 38975874 PMC11528210

[36] M. G. Fakih , L. Salvatore , T. Esaki , et al., “Sotorasib Plus Panitumumab in Refractory Colorectal Cancer With Mutated KRAS G12C,” New England Journal of Medicine 389, no. 23 (2023): 2125–2139, 10.1056/nejmoa2308795. 37870968

[37] R. Yaeger , J. Weiss , M. S. Pelster , et al., “Adagrasib With or Without Cetuximab in Colorectal Cancer With Mutated KRAS G12C,” New England Journal of Medicine 388, no. 1 (2022): 44–54, 10.1056/nejmoa2212419. 36546659 PMC9908297

[38] V. Amodio , R. Yaeger , P. Arcella , et al., “EGFR Blockade Reverts Resistance to KRASG12C Inhibition in Colorectal Cancer,” Cancer Discovery 10, no. 8 (2020): 1129–1139, 10.1158/2159-8290.cd-20-0187. 32430388 PMC7416460

[39] J. Feng , Z. Hu , X. Xia , et al., “Feedback Activation of EGFR/Wild‐Type RAS Signaling Axis Limits KRASG12D Inhibitor Efficacy in KRASG12D‐Mutated Colorectal Cancer,” Oncogene 42, no. 20 (2023): 1620–1633, 10.1038/s41388-023-02676-9. 37020035 PMC10181928

[40] A. Singhal , B. T. Li , and E. M. O’Reilly , “Targeting KRAS in Cancer,” Nature Medicine 30, no. 4 (2024): 969–983, 10.1038/s41591-024-02903-0. PMC11845254 38637634

[41] N. Perurena , L. Situ , and K. Cichowski , “Combinatorial Strategies to Target RAS‐Driven Cancers,” Nature Reviews Cancer 24, no. 5 (2024): 316–337, 10.1038/s41568-024-00679-6. 38627557

[42] M. B. Ryan , O. Coker , A. Sorokin , et al., “KRASG12C‐Independent Feedback Activation of Wild‐Type RAS Constrains KRASG12C Inhibitor Efficacy,” Cell Reports 39, no. 12 (2022): 110993, 10.1016/j.celrep.2022.110993. 35732135 PMC9809542

[43] L. Guo , W. Shao , C. Zhou , et al., “Neratinib for HER2‐Positive Breast Cancer With an Overlooked Option,” Molecular Medicine 29, no. 1 (2023): 134, 10.1186/s10020-023-00736-0. 37803271 PMC10559443

