# Supplementary material for: Pan‐ERBB Inhibitors Synergize With KRAS Inhibitors in Rectal Cancer
Source: United European Gastroenterol J. 2025 Sep 16;13(9):1690–702. doi: 10.1002/ueg2.70086 (PMC12605991; doi:10.1002/ueg2.70086)
Supplement: Supplementary file 1 — Supporting Information S1 [file UEG2-13-1690-s003.docx]

**Material and Methods (Supplemental)**

*Compounds*

The selected drug library (10 mM stock solutions), Sotorasib (AMG-510) (#S8830), and RMC-6236 (#E1597) were obtained from Selleckchem (Cologne, Germany). Neratinib (HY-32721), Poziotinib (HY-15730), and BI-2865 (HY-153724) were sourced from MedChemExpress (Monmouth Junction, NJ, USA). All compounds were dissolved in DMSO.

*Statistics*

Unless otherwise specified, experiments were performed in biological triplicates. The Student’s t-test was applied for comparisons between two groups. Figures display the mean and standard deviation (SD). Data analysis was performed using GraphPad Prism (version 10.1.12, Boston, CA, USA). Bliss, ZIP, and HSA synergy scores and their confidence intervals were calculated using the SynergyFinder tool [1].

*Combinatorial Drug Screen*

SW837 and SW1463 cells were seeded at densities of 3,000 and 5,000 cells/well, respectively, in white 96-well plates (#136101; Thermo Scientific™, Waltham, MA, USA). After 24 hours, cells were treated with a compound library comprising 126 drugs. Experiments were conducted in technical duplicates. A manual liquid handling pin tool (#AFIX96FP; V&P Scientific, San Diego, CA, USA) delivered 0.1 µl of each drug in a 7-point dilution series (3-fold dilutions, highest concentration 10 µM). Library drugs were combined with 0.1 µl of Sotorasib at a fixed concentration of 50 nM or DMSO (final DMSO concentration: 0.2%). After 72 hours of treatment, cell viability was assessed using 25 µL of CellTiter-Glo® Luminescent Assay (#G7573; Promega, Walldorf, Germany). Samples were incubated on an orbital shaker for 20 minutes, and luminescence was measured with a VICTOR™ X4 2030-0040 Multilabel Plate Reader (PerkinElmer Cellular Technologies Germany GmbH, Hamburg, Germany). Dose–response curves were analyzed to determine half-maximal growth inhibitory concentrations (GI50) and area under the curve (AUC) using the R package grmetrics [2]. A drug combination was classified as a hit if ΔAUC < −0.1. Drug response curves exhibiting significant deviations between replicates were excluded. The procedure was exactly the same for the long-term pre-treated SW1463 cells except of the usage of a larger library with 178 drugs. Protein targets of the screening hits were analyzed with a STRING network analysis (<https://string-db.org/>) [3] .

*Cell Number Quantification Over Time*

SW1463 cells were seeded at a density of 25,000 cells/well in 24-well plates. After 24 hours, 50 nM Sotorasib was administered and replenished every 2–3 days. Cells were harvested by 5 minutes incubation in 0.25% Trypsin–EDTA on days 1, 3, 6, 8, 10, and 13. Viable cell counts were obtained, excluding dead cells using 4 g/l Trypan Blue (#1117320025, Merck Millipore) staining.

*Clonogenic Assay*

Cells were seeded in 24-well plates at densities of 5,000 cells/well for GOE-READ139, 10,000 cells/well for SW837, and 25,000 cells/well for SW1463. After 24 hours, cells were treated with individual drugs or combinations. Media were replaced after 3 days. Depending on the cell line, wells were washed with PBS and stained with 0.2% Crystal Violet solution (#T123.3; Carl Roth GmbH + Co. KG, Karlsruhe, Germany) for 10 minutes on an orbital shaker at room temperature. After staining, cells were washed twice with ddH2O, dried, and scanned at 600 dpi. For illustration, images were flipped horizontally. Crystal Violet dye was solubilized in a 1% SDS solution (#CN30.3; Carl Roth GmbH + Co. KG), and absorbance was measured at 570 nm using a VICTOR™ X4 2030-0040 Multilabel Plate Reader.

*In Situ Resistance Assay*

The procedure followed the method described by Sealover et al. [4]. A total of 250 GOE-READ139 and 250 SW837 cells were seeded per well in 96-well plates and cultured in 150 μL of the respective medium. Treatments, either single or combinations, were applied 24 hours post-seeding. Medium and treatments were renewed weekly, and confluence was measured using the Incucyte® SX5 Live-Cell Analysis System (Sartorius, Göttingen, Germany) by capturing five images per well with the standard Incucyte®live-cell analysis Software. Wells that achieved a confluence greater than 50% were considered to have outgrown cell cultures. Kaplan-Meier curves were generated in GraphPad Prism. An outgrown well regarded as an event. The experiment was performed in five technical replicates per condition and repeated in three biological replicates. All biological and technical replicates were analyzed without averaging, comparing the complete distributions between mono- and combination treatment.

*mRNA-Sequencing, Gene Set Enrichment Analysis, and transcription factor prediction*

SW1463 cells were cultured in T25 flasks. RNA extraction was performed using the RNeasy® Mini Kit (Qiagen, #74106). RNA was dissolved in RNase-free water and sequenced at Novogene (Cambridge, UK). Quality control was conducted using FastQC. Reads were aligned with the STAR aligner (version v2.7.11) based on the GRCh38 genome and quantified using Salmon (version v1.10.0). Differential expression analysis was performed with DESeq2 (1.36.0), excluding genes with fewer than 10 reads. Regularized logarithmic count data were used for Gene Set Enrichment Analysis (GSEA) implemented in GSEA software (v4.3.2) [5]  or the GeneTrail 3.2 [6]. Gene sets were obtained from MsigDB v2023.1.Hs.

To predict transcription factor potentially regulating *ERBB2* and *ERBB3* gene expression, we utilized FIMO (Find Individual Motif Occurrences; version 5.5.8) [7]. The upstream genomic region of 2,000 base pairs relative to the TSS of *ERBB2* and *ERBB3* was used for motif scanning with FIMO. Predicted transcription factor binding sites with a p-value threshold of < 0.0001 were retained for further analysis. From the ChEA3 database [8] , we extracted the top 150 predicted transcription factors with lowest arbitrary ChEA3 score of the gene list with *ERBB2* and *ERBB3.* These lists were intersected with all significantly upregulated genes after 24 hours in SW1463 cells (log₂ fold change > 1, p-value < 0.01) and overlaps were presented in a Venn diagram.

*Protein Extraction and Western Blotting*

Cells were seeded in 10 cm dishes, and treatments were applied 24 hours later, with treatments renewed as specified. After treatment, cells were washed with ice-cold PBS and lysed using RIPA buffer (50 mM Tris, 105 mM NaCl, 0.5% Na-deoxycholate, 1% NP-4, 2 mM EDTA, pH 8.0) supplemented with PhosSTOP (#4906837001; Roche Diagnostics) and Protease Inhibitor (#11836170001; Roche Diagnostics). Protein lysates were sonicated in an ultrasonic bath, and concentrations were determined using the Pierce™ BCA Protein Assay Kit (#23225, Thermo Fisher). Samples were mixed with 5 x Lämmli buffer, heated to 95°C for 5 minutes, and 20 μg of protein was loaded onto 10% and 15% Bis-Tris gels for resolution at 20 mA per gel. Proteins were transferred to PVDF membranes (#200T.1; Carl Roth GmbH + Co. KG) using semi-dry blotting or wet blotting (Criterion™ blotter, Bio-Rad) for HER2 and HER3 visualization. Membranes were blocked for 2 hours in 5% milk in Tris-buffered saline with 0.1% Tween® 20 detergent and probed with primary antibodies (Table S3) in 5% milk or BSA overnight at 4°C. Membranes were incubated with secondary antibodies diluted 1:30,000 in 5% milk for 2 hours. Detection was performed using Immobilon® Forte Western HRP substrate (#WBLUF0500; Merck Millipore) and a CCD camera system (LAS 4000mini; GE Healthcare). Signal intensities were quantified with ImageJ (v1.53k). The following antibodies were used:

| **antibody** | **Catalog #** | **Vendor** | **RRID** | **dilution** | **diluent** |
| --- | --- | --- | --- | --- | --- |
| HER3/ErbB3 (D22C5) | #12708 | Cell Signaling | AB_2721919 | 1 : 500 | 5% BSA |
| HER2/ErbB2 (D8F12) | #4290 | Cell Signaling | AB_10557104 | 1 : 500 | 5% BSA |
| Phospho-Akt (Ser473) (D9E) | #4060 | Cell Signaling | AB_2315049 | 1 : 1000 | 5% BSA |
| c-Myc (D84C12) | #5605 | Cell Signaling | AB_1903938 | 1 : 1000 | 5% BSA |
| Akt (pan) (C67E7) | #4691 | Cell Signaling | AB_915783 | 1 : 1000 | 5% BSA |
| anti-KRAS | #WH0003845M1 | Sigma-Aldrich Chemie | AB_1842235 | 1 : 500 | 5% BSA |
| Phospho-p44/42 MAPK (Erk1/2) (Thr202/Tyr204) (D13.14.4E) | #4370S | Cell Signaling | AB_2315112 | 1 : 2000 | 5% milk |
| p44/42 MAPK (Erk 1/2) (137F5) | #4695S | Cell Signaling | AB_10693601 | 1 : 2000 | 5% milk |
| Anti-Cleaved PARP1 antibody [E51] | #ab32064 | abcam | AB_777102 | 1 : 1000 | 5% BSA |
| anti-HSP90 alpha/beta (F-8) | #sc-13119 | Santa Cruz | AB_675659 | 1 : 1000 | 5% milk |
| Phospho-EGF receptor (Tyr1068) | #2234 | Cell Signaling | AB_331701 | 1 : 1000 | 5% BSA |
| EGF Receptor (D38B1) | #4267 | Cell Signaling | AB_2246311 | 1 : 1000 | 5% BSA |
| Mouse Anti-Vinculin Monoclonal Antibody, Unconjugated | #V9131 | Sigma-Aldrich | AB_477629 | 1 : 1000 | 5% milk |

*Drug response profiling of cell lines and PDOs*

To assess the viability and growth of SW1463 (5,000 cells/well) and SW837 (3,000 cells/well) cells were seeded in 96-well white plate (#136101; Thermo Fisher) in 100 μL medium. After 24 hours at 37°C in 5% CO2, cells were treated with 7-point Sotorasib dilutions. Cell viability was assessed after 72 hours using 25 μL of CellTiter-Glo® Luminescent Assay (#G7573; Promega). Luminescence was measured with a VICTOR™ X4 2030-0040 Multilabel Plate Reader.

To assess viability upon treatment of PDOs, a single-cell resuspension was prepared for seeding. The organoids were mechanically disrupted followed by incubation in 3 mL Cell Recovery Solution (#354253, Corning) for 10 minutes on ice, followed by centrifugation. The cells were incubated in 1 mL TrypLE Express Enzyme (#12605028; Gibco) supplemented with 10 μg·mL−1 DNAse I (#D5025; Sigma-Aldrich) and 10 μM Rho Kinase Inhibitor at 37°C for 8 minutes. After centrifugation, the cells were resuspended in PDO medium and were counted. 1250 cells were seeded as 10 µl domes of GeltrexTM in white 96-well plates and in transparent 48-well plates. We allowed PDO formation for 7 days and then applied treatment as 4x4 dilution matrix for 72 hours. Cell viability was assessed using 25 μL of CellTiter-Glo® Luminescent Assay with 10 minutes of gentle shaking followed by 20 minutes incubation at room temperature in the dark and measured on the VICTOR™ X4 plate reader. Images were taken from PDOs in 48-well plate with a Leica DM IL LED microscope.

*Protein expression analysis by Liquid-chromatography-Mass Spectrometry (LC-MS)*

For proteomics, SW1463 cells were seeded in 10 cm dishes. After 24 hours, they were treated with 20 nM Sotorasib and 20 nM Neratinib (0.1 % DMSO). The cells were detached by incubation in 1 mL Trypsin/EDTA and subsequent addition of 10 ml Leibowitz-15 medium. The cells were washed with 10 mL ice-cold PBS once and the pellet was snap-frozen in liquid nitrogen and stored at-80°C. Samples were lysed in 1% SDS, 100 mM HEPES in BeatBox and tryptically digested by SP3 on Resyn Amine Beads in Kingfisher Duo Prime. 800 ng equivalent loaded was measured with Thermo Exploris 480. Spectronaut (v19.7) was used for protein identification and spectral library generation against the UniProtKB human reference proteome (v8.2023) with default settings and a 54-protein in-house contaminant database (1% FDR). DIA quantification included up to 6 fragments per peptide, 10 peptides per protein, dynamic retention time alignment, dynamic mass recalibration, and quartile normalization (1% FDR). The raw data was logarithmic transformed (base 2) and statistically compared with limma package (v3.62) in R. Gene set enrichment was computed with GSEA software.

**References: Material and Methods**

[1] Ianevski A, Giri AK, Aittokallio T. SynergyFinder 2.0: visual analytics of multi-drug combination synergies. Nucleic Acids Res 2020;48:W488–93. <https://doi.org/10.1093/nar/gkaa216>.

[2] Clark NA, Hafner M, Kouril M, Williams EH, Muhlich JL, Pilarczyk M, Niepel M, Sorger PK, Medvedovic M. GRcalculator: an online tool for calculating and mining dose–response data. Bmc Cancer 2017;17:698. <https://doi.org/10.1186/s12885-017-3689-3>.

[3] Szklarczyk D, Gable AL, Lyon D, Junge A, Wyder S, Huerta-Cepas J, Simonovic M, Doncheva NT, Morris JH, Bork P, Jensen LJ, von Mering C. STRING v11: protein–protein association networks with increased coverage, supporting functional discovery in genome-wide experimental datasets. Nucleic Acids Res 2018;47:gky1131. <https://doi.org/10.1093/nar/gky1131>.

[4] Sealover NE, Theard PT, Hughes JM, Linke AJ, Daley BR, Kortum RL. In situ modeling of acquired resistance to RTK/RAS-pathway-targeted therapies. IScience 2024;27:108711. <https://doi.org/10.1016/j.isci.2023.108711>.

[5] Subramanian A, Tamayo P, Mootha VK, Mukherjee S, Ebert BL, Gillette MA, Paulovich A, Pomeroy SL, Golub TR, Lander ES, Mesirov JP. Gene set enrichment analysis: a knowledge-based approach for interpreting genome-wide expression profiles. P Natl Acad Sci Usa 2005;102:15545–50. <https://doi.org/10.1073/pnas.0506580102>.

[6] Gerstner N, Kehl T, Lenhof K, Müller A, Mayer C, Eckhart L, Grammes NL, Diener C, Hart M, Hahn O, Walter J, Wyss-Coray T, Meese E, Keller A, Lenhof H-P. GeneTrail 3: advanced high-throughput enrichment analysis. Nucleic Acids Res 2020;48:gkaa306-. <https://doi.org/10.1093/nar/gkaa306>.

[7] Grant CE, Bailey TL, Noble WS. FIMO: scanning for occurrences of a given motif. Bioinformatics 2011;27:1017–8. <https://doi.org/10.1093/bioinformatics/btr064>.

[8] Keenan AB, Torre D, Lachmann A, Leong AK, Wojciechowicz ML, Utti V, Jagodnik KM, Kropiwnicki E, Wang Z, Ma’ayan A. ChEA3: transcription factor enrichment analysis by orthogonal omics integration. Nucleic Acids Res 2019;47:W212–24. <https://doi.org/10.1093/nar/gkz446>.
