# Supplementary material for: Pan‐ERBB Inhibitors Synergize With KRAS Inhibitors in Rectal Cancer
Source: United European Gastroenterol J. 2025 Sep 16;13(9):1690–702. doi: 10.1002/ueg2.70086 (PMC12605991; doi:10.1002/ueg2.70086)
Supplement: Supplementary file 2 — Supporting Information S2 [file UEG2-13-1690-s007.docx]

Buchloh_Supplementary_Table_1R1: Captions: Goettingen rectal cancer cohort

Buchloh_Supplementary_Table_2R1: Captions: Patient-derived cell lines

Buchloh_Supplementary_Table_3R1: Captions: Sotorasib RNA-seqencing

Buchloh_Supplementary_Table_4R1: Captions: Drug screening data

Buchloh_Supplementary_Table_5R1: Captions: Proteomics data

Buchloh_Supplemental_Figure_1: Captions: KRAS mutations and clinical response

Buchloh_Supplemental_Figure_2: Captions: Treatment and DFS

Buchloh_Supplemental_Figure_3: Captions: EGFRi and long-term adaptation

Buchloh_Supplemental_Figure_4: Captions: Potential ERBB2 and ERBB3 regulators
